# Supplementary material for: Empirical methods for controlling false positives and estimating confidence in ChIP-Seq peaks
Source: BMC Bioinformatics. 2008 Dec 5;9:523. doi: 10.1186/1471-2105-9-523 (PMC2628906; doi:10.1186/1471-2105-9-523)
Supplement: Additional File 1 — A variety of html documents from the USeq web site detailing the available applications, their best usage, output file type descriptions, command line menus, etc. [file 1471-2105-9-523-S1.zip › USeqUserGuides/JavaDoc.zip!/JavaDoc/edu/utah/seq/parsers/ElandExportLine.html]

ElandExportLine


|  |  |  |
| --- | --- | --- |
| **Overview** \| **Package** \| **Class** \| **Use** \| **Tree** \| **Deprecated** \| **Index** \| **Help** | |  |
| **PREV CLASS**   **NEXT CLASS** | **FRAMES**    **NO FRAMES**     **All Classes** |  |
| SUMMARY: NESTED \| FIELD \| CONSTR \| METHOD | DETAIL: FIELD \| CONSTR \| METHOD |  |


---


## edu.utah.seq.parsers Class ElandExportLine

```
java.lang.Object
  edu.utah.seq.parsers.ElandExportLine
```

---

``` public class ElandExportLine extends java.lang.Object ```

---

| **Field Summary** | |
| --- | --- |
| `static java.util.HashMap<java.lang.String,java.lang.Integer>` | `asciiValue` |


**Constructor Summary**

`ElandExportLine(java.lang.String line,
int[][] gatc)`
  
          Stripped constructor/ method for ElandSequenceParser

`ElandExportLine(java.lang.String line,
int offSet,
boolean parseIt)`
  
          For parsing a stand alone Eland run.

`ElandExportLine(java.lang.String line,
int offSet,
float minScore)`
  
          Parses an Eland Extended output line, will shift the stranded position by the offSet.


| **Method Summary** | |
| --- | --- |
| `static java.util.HashMap<java.lang.String,java.lang.Integer>` | `asciScore()`             Returns a map of asci text character to it's associated Solexa base quality score. |
| `static int[]` | `convertScores(java.lang.String seqQual)`             Converts the ascii quality scores to numeric scores. |
| `java.lang.String` | `fetchChromStrandName()` |
| `Point` | `fetchPoint()` |
| `int` | `fetchReadLength()` |
| `java.lang.String` | `getChromosome()` |
| `int` | `getPosition()` |
| `float` | `getScore()` |
| `boolean` | `parsed()` |
| `void` | `setChromosome(java.lang.String chromosome)` |
| `void` | `setPosition(int position)` |
| `void` | `setScore(float score)` |

**Methods inherited from class java.lang.Object**

`equals, getClass, hashCode, notify, notifyAll, toString, wait, wait, wait`

**Field Detail**

### asciiValue

```
public static final java.util.HashMap<java.lang.String,java.lang.Integer> asciiValue
```


**Constructor Detail**

### ElandExportLine

```
public ElandExportLine(java.lang.String line,
                       int offSet,
                       float minScore)
```

:   Parses an Eland Extended output line, will shift the stranded position by the offSet.
    HWI-EAS240 FC2087UAAXX 8 1 237 408 TACACATGAATTCAACTTAAATTCCTTGTTAAAATT ZZZZZZZZZZZZZZZZZZZZZZZZZZZZZZVUUVVU chr6.fasta 147077758 F 36 73 Y
    HWI-EAS240 FC2087UAAXX 8 1 311 379 TACTTGGTTAAGTAGGATGTTATTCTGCTTCTACAC ZZZZZZZZZZZZZZZZZZZZZZZZVZRZZZUVLLLL chr3.fasta 154412997 R 36 65 Y
    HWI-EAS240 FC2087UAAXX 8 1 219 459 TACAACATGTACAAGCCTAAATCCTTTTAGCCAGAG ZZZZZZZZZZZZZZZZZZZZZZZZZZZZZJUOSOUQ NM Y
    HWI-EAS240 FC2087UAAXX 8 1 582 773 TCATGTTAAAGTGATTGTGATGTTTGAAAACCAATG ZZZZZZZZZZZZZZZZZZZZZVZZZZJZZZSUSVVG chr6.fasta 123151134 R 36 54 Y
    HWI-EAS240 FC2087UAAXX 8 1 820 387 TAAATTGTAACATAAAATTCTTATGAAATTACCTCA ZKZZZZZZZZZZZZZZZZZZZZZZIZOZZZOODOOH chr16.fasta 58060756 R 36 39 N
    Note, Reverse match annotation are relative to the reverse (seq is reverse, quality reversed, base changes are base changes needed to match consensus,
    the sequence given is the oligo sequence not the reference, position is in forward space though.)
    Only lines that have passed the qc flag and have an alignment will be parsed so check the parsed() before using.

---


### ElandExportLine

```
public ElandExportLine(java.lang.String line,
                       int[][] gatc)
```

:   Stripped constructor/ method for ElandSequenceParser

---


### ElandExportLine

```
public ElandExportLine(java.lang.String line,
                       int offSet,
                       boolean parseIt)
```

:   For parsing a stand alone Eland run.
    Score set to 1
    /home/solexa/Hg17SpikeIns/reads.txt-1 ttccactgagcctgctccagaactt U2 0 0 1 chr1 53226799 F .. 23A 25C


**Method Detail**

### convertScores

```
public static int[] convertScores(java.lang.String seqQual)
```

:   Converts the ascii quality scores to numeric scores.

---


### asciScore

```
public static java.util.HashMap<java.lang.String,java.lang.Integer> asciScore()
```

:   Returns a map of asci text character to it's associated Solexa base quality score.

---


### parsed

```
public boolean parsed()
```

---


### fetchChromStrandName

```
public java.lang.String fetchChromStrandName()
```

---


### fetchReadLength

```
public int fetchReadLength()
```

---


### fetchPoint

```
public Point fetchPoint()
```

---


### getChromosome

```
public java.lang.String getChromosome()
```

---


### setChromosome

```
public void setChromosome(java.lang.String chromosome)
```

---


### getPosition

```
public int getPosition()
```

---


### setPosition

```
public void setPosition(int position)
```

---


### getScore

```
public float getScore()
```

---


### setScore

```
public void setScore(float score)
```


---


|  |  |  |
| --- | --- | --- |
| **Overview** \| **Package** \| **Class** \| **Use** \| **Tree** \| **Deprecated** \| **Index** \| **Help** | |  |
| **PREV CLASS**   **NEXT CLASS** | **FRAMES**    **NO FRAMES**     **All Classes** |  |
| SUMMARY: NESTED \| FIELD \| CONSTR \| METHOD | DETAIL: FIELD \| CONSTR \| METHOD |  |


---
